# Exploring the Relationship Between White Matter Tracts and Resting-State Functional Language Lateralization Index

**DOI:** 10.1162/nol_a_00167

**Published:** 2025-06-18

**Authors:** Marie-Ève Desjardins, Karine Marcotte, Xanthy Lajoie, Christophe Bedetti, Bérengère Houzé, Abdelali Filali-Mouhim, Arnaud Boré, Maxime Descoteaux, François Rheault, Simona Maria Brambati

**Affiliations:** Centre de Recherche de l’Institut Universitaire de Gériatrie de Montréal, Montréal, Québec, Canada; Département de Psychologie, Université de Montréal, Montréal, Québec, Canada; Centre de recherche du Centre intégré universitaire de santé et de services sociaux du Nord-de-l’Île-de-Montréal, Montréal, Québec, Canada; École d’orthophonie et d’audiologie, Université de Montréal, Montréal, Québec, Canada; Département d’informatique, Université de Sherbrooke, Sherbrooke, Québec, Canada

**Keywords:** diffusion tensor imaging, functional magnetic resonance imaging (fMRI), language lateralization, machine learning, resting-state, tractography

## Abstract

Resting-state functional magnetic resonance imaging (rs-fMRI) enables the evaluation of the language network and is particularly useful for measuring language lateralization with minimal participant effort and methodological biases (e.g., no language task execution or selection). Tractography using diffusion MRI (dMRI) provides complementary information on language-associated white matter bundles. Some structural white matter measures of the left or right hemisphere have been related to the functional language lateralization index (LI) and allow a better understanding of this network. This study utilizes tractography to identify white matter structural predictors of LI from a single hemisphere, employing linear regression and random forest models. Rs-fMRI and dMRI data from 618 healthy subjects of the Human Connectome Project were used to link LI to micro- and macro-structural measures of the arcuate fasciculi, the inferior longitudinal fasciculi, the frontal aslant tracts and sections of the corpus callosum. Results suggest a possible relationship between micro- and macro-structural measures of white matter tracts, and functional language lateralization measured in resting-state. However, the identified predictors are not sufficiently representative to be considered proxies for functional language lateralization. In conclusion, both micro- and macro-structural white matter characteristics as well as both left and right hemispheres are important to consider, but are not sufficient on their own, when investigating the relationship between brain structures and functional language lateralization.

## INTRODUCTION

Although the language network presents a bilateral organization, language functions in most individuals mainly rely on regions of the left hemisphere (Knecht et al., 2000). Many researchers have highlighted the importance and complexity of studying the degree of left-lateralization in language, which varies across individuals (Mazoyer et al., 2014). For example, brain damage in the left hemisphere could cause more severe acquired language deficits in patients with more left-lateralized language (Anglade et al., 2014; Knecht et al., 2002; Turkeltaub, 2019). A better understanding of structures underlying this functional lateralization would help guide clinical studies, for example, in predicting language deficits caused by a stroke or a tumor. Thus, structural markers of language lateralization based on single-hemisphere characteristics in healthy subjects could be applied to patients with acquired left- or right-hemisphere lesions, in whom the functional signal and the language lateralization based on a ratio between the two hemispheres may be biased. This could represent a first necessary step to develop future clinical studies exploring the role of language lateralization in patients’ language recovery when functional data are not available or possible to acquire.

Historically, the gold standard for assessing language lateralization was the Wada test. The invasive nature of this technique (X-ray dye injection and use of anesthetics) has reduced its popularity and has motivated the use of fMRI in the measurement of language lateralization, either in healthy or clinical populations, particularly in epilepsy patients (Adcock et al., 2003; Arora et al., 2009; Detre, 2004; Liégeois et al., 2002; Sabbah et al., 2003; Woermann et al., 2003). Several studies have explored and subsequently supported the validity of task-based fMRI (tb-fMRI) compared to the WADA test for measuring language lateralization (Bauer et al., 2014; Dym et al., 2011). However, despite the advantages provided by tb-fMRI over the Wada test, many researchers have criticized the high variability in the LI, which depends on the chosen language task paradigm (for a comprehensive review on this topic, see Bradshaw et al., 2017b). The use of task-free fMRI protocols, such as rs-fMRI, could provide a valid alternative approach to overcome some methodological limitations of tb-fMRI. Previous studies have confirmed the validity of the LI based on rs-fMRI data by comparing it to the Wada test (96% agreement; DeSalvo et al., 2016) and to the tb-fMRI LI (up to 85% correlation with functional connectivity characteristics; Smitha et al., 2017). Among others, seed-based functional connectivity using the left inferior frontal gyrus as a seed can distinguish typical from atypical language lateralization in rs-fMRI (Wang et al., 2019).

One unresolved question is whether the LI obtained with rs-fMRI approaches is related to the morphological characteristics of the brain structures involved in the language network. In particular, recent evidence has pointed out the possible role of white matter connectivity in the emergence of language laterality (Al Busaidi et al., 2023; Andrulyte et al., 2024; Häberling et al., 2011; James et al., 2015; Karpychev et al., 2022; Perlaki et al., 2013; Powell et al., 2006; Propper et al., 2010; Sreedharan et al., 2015; Vassal et al., 2016; Vernooij et al., 2007; Westerhausen et al., 2006; Yazbek et al., 2021; Zahnert et al., 2023). These studies have focused on the possible link between laterality of the functional language network and the micro- and macrostructural characteristics (volume, density, surface areas, fractional anisotropy, mean diffusivity, etc.) of the fiber bundles connecting the core regions of the network itself, based on dMRI. Because of its role in the language network, the arcuate fasciculus (AF) is one of the most studied fiber bundles in relation to language laterality. Results suggest a possible relationship between language lateralization measured with tb-fMRI and macrostructural characteristics of the left and right AF, such as the density or volume (James et al., 2015; Propper et al., 2010; Sreedharan et al., 2015; Vassal et al., 2016). However, these results were not consistently replicated across studies (for an example of alternative results, see Al Busaidi et al., 2023). From a microstructural perspective, in right-handed individuals, studies suggest a possible link between the tb-fMRI LI and the fractional anisotropy of the right AF (Yazbek et al., 2021), but not necessarily with the left AF (Piervincenzi et al., 2016; Yazbek et al., 2021). The corpus callosum (CC) is also of interest considering its function of interhemispheric communication. Independent studies have shown that greater volume and surface area of the whole CC or its segments are associated with more left-lateralized language (Josse et al., 2008; Karpychev et al., 2022). On the other hand, studies on microstructural characteristics of CC have focused on heterogeneous set of variables, and the results remain generally inconclusive (Häberling et al., 2011; Karpychev et al., 2022; Perlaki et al., 2013; Westerhausen et al., 2006). Only a few studies have addressed the inferior longitudinal fasciculus (ILF) and the frontal aslant tract (FAT), and although no consistent pattern emerges, studies emphasize the importance of studying these fiber bundles as they are part of language networks (Al Busaidi et al., 2023; Andrulyte et al., 2024; James et al., 2015). In summary, these studies have provided some evidence supporting the hypothesis that the characteristics of these fiber bundles can underlie language lateralization. However, it is hard to infer a coherent picture from these results. Importantly, some limitations and methodological choices must be considered. For example, most studies are based on small sample sizes, offering only limited statistical power to explore multiple bundles, and therefore sometimes focus on a single or few white matter tracts and a reduced number of diffusion metrics (which vary across studies).

Current big data initiatives, and more specifically the Human Connectome Project (HCP), provide access to a larger sample of multimodal dMRI and fMRI data and offer the opportunity to explore more deeply the relationship between structural characteristics and language lateralization, as suggested in a previous study (Lee et al., 2013). Recently, Andrulyte and colleagues (2024) addressed this question with the HCP database. Results showed that both microstructural architecture (regional white matter quantitative anisotropy) and macrostructural geometric features of white matter tracts (such as CC, inferior fronto-occipital fasciculus [IFOF] and forceps minor) play a role in language lateralization. While providing valuable information on a wide range of both tracts and metrics, this study calculated LI (or laterality quotient, LQ as called in their article) based on a comprehension task. The LQ may therefore be influenced by the choice of the language task used in the scanner (Bradshaw et al., 2017b). Resting-state methods, however, are attracting growing interest to overcome this limitation, particularly in clinical contexts, as rs-fMRI does not require specific stimuli equipment or active patient participation (Takamura & Hanakawa, 2017). Thus, LI based on rs-fMRI can offer a valuable complementary tool to try to understand the relationship between LI and white matter bundle characteristics.

In the present article, we aim to investigate whether white matter bundle characteristics of each hemisphere separately (and not as a ratio) can represent indicators of LI. More precisely, with a group of 618 healthy young adults, our objective is to explore and compare models using a wide range of micro- and macrostructural characteristics of white matter bundles (from the combined and separate left and right hemispheres) to predict the LI calculated with a seed-based approach using a core seed in the production network. This question is addressed in three ways using univariate linear regressions, regularized multiple regressions, and a machine learning approach based on a set of trees. According to the literature, the prediction of LI is based on micro- and macrostructural characteristics of the AF, ILF, FAT, as well as the CC. Rs-fMRI and dMRI data are issued from the HCP S1200 release database (Van Essen et al., 2012).

## MATERIALS AND METHODS

### Participants

Six hundred and eighteen subjects (320 women and 298 men; 30 left-handed, 57 ambidextrous, 531 right-handed; mean age 28 ± 4 yr; mean education 15 ± 2 yr) from the HCP database were included in the study. The Washington University—University of Minnesota Consortium of the HCP (WU-Minn HCP) dataset includes, among others, brain imaging (high quality structural MRI, high angular resolution dMRI, and rs-fMRI) of 1,200 healthy young adult twins and non-twin siblings aged 22 to 36 years old (Van Essen et al., 2012). The filters applied to the S1200 dataset were the following: We included all participants with four completed resting-state scans and six completed dMRI acquisitions, and who belonged to the most recent data release; we excluded subjects with quality control issues. Data acquisition and preprocessing are included in the 1200 Subjects release and are summarized below (see the 1200 Subjects Data Release Reference Manual, available at https://www.humanconnectome.org/storage/app/media/documentation/s1200/HCP_S1200_Release_Reference_Manual.pdf, for full technical documentation).

The WU-Minn HCP Open Access Data Use Form was filled out. This included an agreement to comply with institutional regulations. The documentation to access the restricted data and engage the team to confidentiality was signed. Although not mandatory to access the HCP database, the approval of our local ethics committee was obtained (CER VN 22-23-08).

### Magnetic Resonance Imaging Acquisition

Anatomical, diffusion and resting-state functional images were acquired with a Siemens Skyra 3T MR scanner with a 32-channel head coil. High-resolution anatomical images were acquired using a T1w-3D MPRAGE, repetition time (TR) = 2,400 ms, echo time (TE) = 2.14 ms, flip angle = 8°, field of view (FOV) = 224 × 224 mm, voxel size = 0.7 mm isotropic. DMRI images were acquired using a spin-echo EPI sequence, TR = 5,520 ms, TE = 89.5 ms, flip angle = 78°, FOV = 210 × 180 mm, voxel size = 1.25 mm isotropic, b-values: 1,000, 2,000, and 3,000 s/mm^2^, approximately 90 diffusion weighting directions plus six b = 0 acquisitions, and a total of 111 slices were acquired to cover the whole brain. Rs-fMRI images were acquired using a gradient-echo EPI sequence, TR = 720 ms, TE = 33.1 ms, flip angle = 52°, FOV = 208 × 180 mm (RO × PE), voxel size = 2 mm isotropic, number of slices = 72. The data consisted of 1,200 volumes for each run for a total of 4,800 volumes for each subject. For the resting-state data acquisition, subjects were asked to lie with eyes open, with “relaxed” fixation on a white cross (on a dark background), to think of nothing in particular and not to fall asleep.

### Preprocessing of MRI Data

Structural and functional imaging data were preprocessed using the HCP MR data preprocessing pipelines (detailed in Glasser et al., 2013). Structural preprocessing pipelines include gradient distortion correction, coregistration and averaging of T1w and T2w runs, field map distortion correction, and Freesurfer (Fischl, 2012) skull stripping. FMRI pipelines included gradient distortion correction, realignment to compensate for subject motion, registration of fMRI data to structural T1w, intensity normalization, normalization to MNI (Montreal Neurological Institute) space, and smoothing.

Diffusion imaging data were preprocessed using the pipeline TractoFlow (tractoflow-2.2.1). Diffusion preprocessing pipeline included normalization of the b0 image intensity, EPI and eddy-current distortions correction, motion and gradient-nonlinearity corrections, and registration of the diffusion data with the structural and brain masking. The final step of this pipeline calculates diffusion metrics and participants’ tractogram, that is, a representation of the anatomical organization of white matter (Theaud et al., 2020).

### Tractometry

From tractograms and reference white matter bundle atlases (https://zenodo.org/records/7562635), RecobundlesX pipeline (rbx_flow-1.1.0; Garyfallidis et al., 2018; Rheault, 2020) isolated the bundles of interest: AF, ILF, FAT, IFOF, and CC segmented in six anterior-posterior parts (CC_Fr_1 : frontal lobe (most anterior part), CC_Fr_2 : frontal lobe (most posterior part), CC_Oc : occipital lobe, CC_Pa : parietal lobe, CC_Pr_Po : pre/post central gyrus, CC_Te : temporal lobe). Finally, the Tractometry pipeline (tractometry_flow-1.0.0 with scilpy-1.6.0; Cousineau et al., 2017) took as inputs the metrics extracted by TractoFlow and the bundles isolated by RecobundlesX, and provided as outputs the data relevant to the project: microstructural features such as fractional anisotropy, radial/axial/mean diffusivity, and apparent fiber density, as well as macrostructural characteristics including length features (average length, span, diameter), shape features (curvature, elongation, irregularity), surface area, and volume (for complete descriptions of macrostructural features, see Yeh, 2020) for all tracts of interest listed above. To serve the purpose of this study, the six segments of the CC was divided in left and right sections using the seven more right subdivisions and seven more left subdivisions of the fifteen subdivisions provided by Tractometry pipeline to form left and right parts of the CC’s segments, respectively. To control for interindividual differences, all left and right hemisphere tract volumes were corrected by dividing it by the total white matter volume (from left and right hemispheres, respectively). Using an adjusted version of Schilling and collaborators (2022) equations, corrections were performed for lengths ([3 * left or right hemisphere total white matter volume of a participant/4pi]^[1/3]) and areas ([left or right hemisphere total white matter volume of a participant/4pi]^[1/2]) features. Note that because CC lengths and area features are based on both left and right parts for each segment (frontals, parietal, pre/post central gyrus, temporal, and occipital), corrections were made using total white matter volume of both hemispheres.

### Quality Assessment

**Figure 1.**
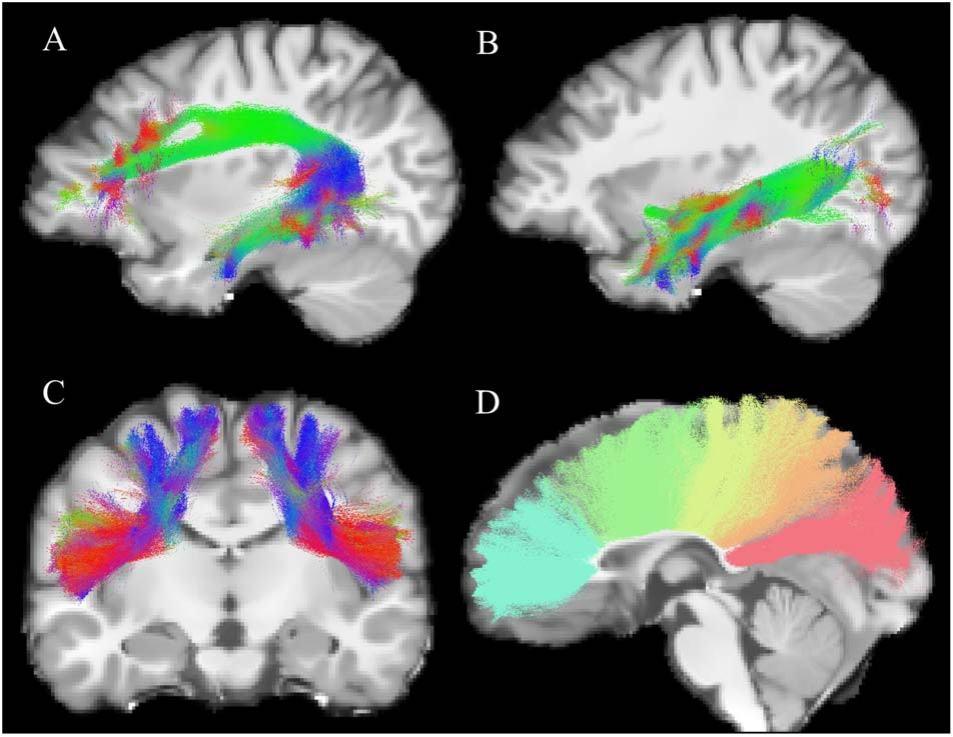
Examples of bundle reconstructions. Reconstruction of the (A) left arcuate fasciculus, (B) left inferior longitudinal fasciculus, (C) left and right frontal aslant tracts (front view), and (D) five segments (frontal anterior, frontal-posterior, pre/post central gyrus, parietal, occipital) of the corpus callosum (left view) from one representative participant.

To ensure quality after the Tractoflow pipeline, a visual inspection was performed on the diffusion data (fractional anisotropy, peaks, and residuals), denoised T1 images, and tissue segmentation images by two independent raters (MED and SMB). A visual quality assurance (shape, size) was performed twice on all tracts of interest after tract reconstruction with RecobundlesX by four raters (MED, SMB, XL, and BH). The visual quality check brought to light some abnormalities in the reconstruction of bundles (low number of streamlines, holes in bundles). When these abnormalities were major and suggested a bad reconstruction, the raters discussed and reached a consensus on whether to keep these data or to exclude them. Through this process, a total of 6.6% of all tracts data were manually excluded from the database (3.4% of the left AF; 1.1% of the right AF; 21% of the left FAT; 18% of the right FAT; 14% of the anterior frontal segment of the CC; 11% of the frontal-posterior segment of the CC; 1% of the pre/post central segment of the CC; 1.5% of the parietal segment of the CC; and 1.6% of the occipital segment of the CC). This quality check identified flaws in the reconstruction of the IFOF and the temporal segment of the CC. In a portion of subjects—17% for the left IFOF, 11% for the right IFOF, and 18% for the CC temporal segment—the algorithm reconstructed only a small number of streamlines (often fewer than 100). Additionally, in 10% of cases for the left IFOF, 5% for the right IFOF, and 13% for the CC temporal segment, no tracts were reconstructed at all. Considering those high rates, it was decided to remove both IFOF and temporal CC segments from analyses. Reconstruction of left and right AF, ILF, FAT as well as the five CC segments was achieved for all participants (see Figure 1 for an example of bundles reconstruction).

### Rs-fMRI Functional Connectivity Analyses

Statistical analyses of rs-fMRI data were performed with Nilearn (Version 0.10.4; https://nilearn.github.io; Nilearn Contributors, 2023). The selection of the seeds for the whole-brain seed-to-voxel connectivity analyses was based on previous work by Battistella and collaborators (2019, 2020) that support its validity in the study of the language network. To represent articulatory (production) subnetwork, because of its recurrent inclusion in language lateralization studies and its support in temporal reliability as well as lateralizing effects in rs-fMRI (Zhu et al., 2014), the main seed was placed in the opercular part of the left inferior frontal gyrus (opIFG; MNI coordinates: *x* = −50, *y* = 8, *z* = 23). Since different seeds could target other language subnetworks (phonological/perception, semantic, and multimodal orthography-to-phonology processes; Battistella et al., 2020), three other seeds were also included and are provided in the Supplementary Materials, available at https://doi.org/10.1162/nol_a_00167. Spheres of 8 mm radius were centered on the chosen coordinates, and the blood-oxygen level dependent (BOLD) signal was extracted from the regions of interest (ROIs) for each participant. For each subject, the average time series from the seed was extracted and used to compute the temporal correlation with all remaining voxels in the brain. During time courses extraction, band-pass filters (0.01 Hz < f < 0.10 Hz) were applied, and BOLD signal from cerebrospinal fluid and the motion correction parameters in the six dimensions were included as regressors. The *r*–Pearson correlation maps of each voxel’s connectivity strength to the ROIs were created. Correlation maps were then converted to z-scores by Fisher’s *r*-to-z transformation.

### Lateralization Index

According to recommendations concerning the choice of LI method (Bradshaw et al., 2017a) and as used in previous resting-state functional language network literature (Battistella et al., 2020), the AveLI toolbox (Matsuo et al., 2012) was used to calculate the LI based on converted connectivity maps of each participant. AveLI is calculated by computing subordinate LI for each positive z-score voxel of the network. By giving greater weight to voxels more correlated to the network, AveLI allows a better control over noise. Another advantage of this method is that AveLI is based on the magnitude rather than the extent of the signal/correlation, making it potentially more suitable for capturing individual variability in language lateralization beyond the binary distinction of typical versus atypical (Bradshaw et al., 2017a). Also, it is threshold independent (reducing bias generated by subjective choices), and is well supported for its reproducibility (Matsuo et al., 2021).

FSLeyes software (McCarthy, 2025) was used to create masks in preparation for AveLI calculations. The masks were defined using the SENtence Supramodal Areas AtlAS (SENSAAS; Labache et al., 2019). From this openly accessible atlas (https://github.com/loiclabache/SENSAAS_brainAtlas/), we selected 18 ROIs corresponding to the core language network (SENT_CORE network). These regions are defined as being significantly involved in language processing during tb-fMRI (including sentence listening, reading, and production) and as being intrinsically connected. This atlas is derived from the Atlas of Intrinsic Connectivity of Homotopic Areas (Joliot et al., 2015), which is specifically designed for resting-state connectivity analyses. It is particularly well-suited for identifying functionally homotopic ROIs, allowing for precise computation of functional asymmetries. The ROIs (18 left, 18 right) were used to create left and right masks separately. The AveLI toolbox utilizes Fisher z-transformed Pearson correlation maps of connectivity strength across all brain voxels, along with left and right masks as inputs. It calculates an average LI by voxel values, applying a weighting based on all positive values.

The following equation, adapted from Matsuo and collaborators (2012), served to calculate subordinate LI:

$$\text{sub}-\text{LI}=\frac{\text{Lz}-\text{Rz}}{\text{Lz}+\text{Rz}}$$

where sub-LI is the subordinate lateralization index and Lz and Rz are the sums of the z-scores at and above zero in the left and right ROIs. All sub-LI were then averaged using this formula (Matsuo et al., 2012):

$$\text{AveLI}=\frac{\Sigma \text{sub}-\text{LI}}{\text{VN}}$$

where AveLI is the average of all sub-LI (and will be referred to as LI hereafter for simplicity), VN is the total number of voxels with positive z-scores within the bilateral ROIs. Thus, the degree of functional language lateralization was calculated for each subject.

### Statistical Analyses

First, our database contained a total of 7% missing data distributed across different variables and participants. These were mainly neuroimaging data and very little demographic data. Those missing data were imputed using rfImpute function as implemented in the R package randomForest (Breiman, 2001). The algorithm began by performing a rough imputation of missing values: for numeric variables, NAs were replaced with the column medians, while for factor variables, they were replaced with the most frequent levels. The randomForest function was then applied to the completed dataset, using the response vector as the outcome. The proximity matrix from the randomForest was used to update the imputation of the NAs. For continuous predictors, the imputed value was the weighted average of the non-missing observations, where the weights were the proximities. This process was iterated five times with 300 trees grown in each iteration. The completed database was then ready for all analyses.

Next, univariate linear regressions were performed using the lm function as implemented in the R package Stats. A regression was calculated for all the 178 demographic (age, sex, education, handedness, and socioeconomic status) and neuroimaging (micro- and macrostructural features of AF, ILF, FAT, and five segments of the CC) data as predictors, and the LI as the predicted outcome. The *p* values for the univariate regressions were corrected for false discovery rate (FDR) using the Benjamini–Hochberg correction (Benjamini & Hochberg, 1995), and were considered significant when *p*_FDR_ < 0.05.

To compare the contribution of each hemisphere independently in the prediction of LI, three regularized multiple regression models were then created using: (1) demographic, left- and right-hemisphere data; (2) demographic and left-hemisphere data; and (3) demographic and right-hemisphere data. Regularized multiple regression prediction models were performed using elastic net regression, a regularized regression method. By adding a constraint in the equation (also known as shrinkage or regularization), this method allows the creation of a linear regression model that is penalized for having too many variables, and performs both variable selection and regularization simultaneously (Zou & Hastie, 2005). The R packages caret (Kuhn, 2008) was used to test a range of possible alpha (the penalty parameter) and lambda (the amount of the shrinkage) values using a 10-fold cross-validation. The selected best values then resulted in a final elastic net model.

In this study, feature selection was conducted by training a model on 70% of the sample while using the remaining 30% as the test set. Using the data from the test sample, the model’s overall performance was reported as an *r*-Pearson correlation between predicted and actual values as well as the root-mean-square error (RMSE), which corresponds to the average difference between the observed known values of the outcome and the predicted value by the models.

Finally, the prediction of the LI was also performed using random forest classification models (Breiman, 2001) as implemented in the ranger R package (Wright & Ziegler, 2017). The predictors were the same demographic and neuroimaging data listed above. Random forest models used bagging (bootstrap aggregating) of decision trees to reduce the variance of single trees and thus improve prediction accuracy compared to a single tree. A set of decision trees were trained on bootstrap samples of the data. At each node of each tree, a random subset of a fixed size was selected from the features, and the one yielding the maximum decrease in Gini index (Breiman, 2001) was chosen for the split. Variable importance was measured by a permutation-based feature importance method and was used to compute a permutation-based *p* value as implemented in Altman and collaborators (2010) for variables selection purpose. The test error of random forest models was estimated on the out-of-bag data, as follows: After each tree has been grown, the inputs that did not participate in the training bootstrap sample were used as a test set, then the average over all trees gave the test error estimate. We used 500 random trees and the default “mtry” (number of variables randomly sampled and tested in each node) parameter. The prediction error was measured by the RMSE. Similarly to regularized multiple regression models described above, three random forest models were created. All statistical analyses were performed using the statistical package R (Version 4.4.1; R Core Team, 2022).

## RESULTS

To meet the objectives of the present study, the results presented are based on LI measured with opIFG seed only; comprehensive results including regressions and models with LI based on the three other seeds can be found in Figures S3–S14 and Tables S1–S9 in the Supplementary Materials.

### LI and Bundle Reconstruction

**Figure 2.**
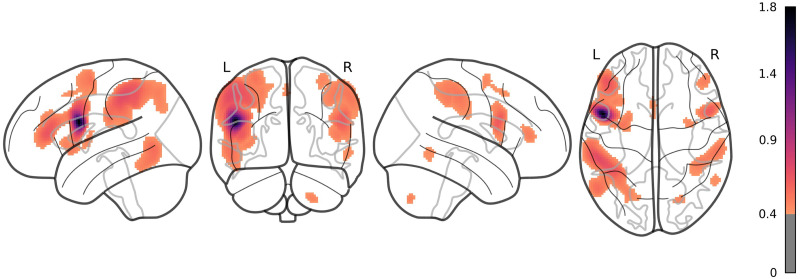
Mean correlation maps (z-scores transformed) of the rs-fMRI language network obtained from a seed in the left inferior frontal gyrus (opIFG). A threshold of *z* = 0.4 was applied to facilitate visualization.

**Figure 3.**
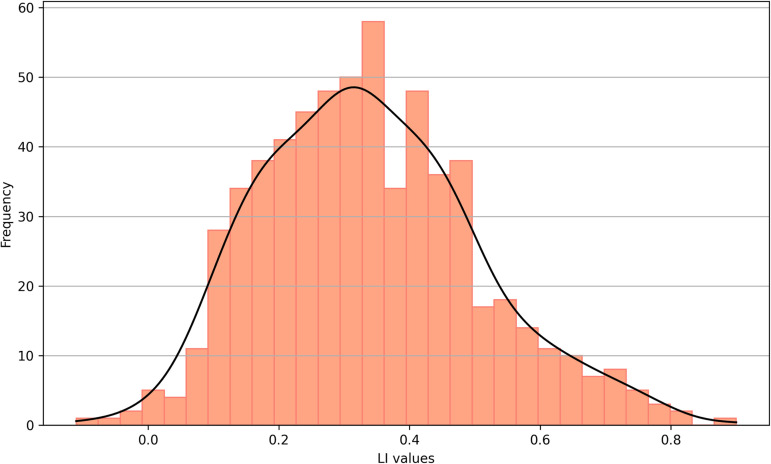
Frequency distribution of functional language lateralization indexes (LI) obtained with the opIFG seed.

Analyses were performed on the 618 participants from the HCP. Patterns of resting-state functional connectivity were successfully isolated in all participants (Figure 2) and LI ranged from −0.11 to 0.90 (*M* = 0.34, *SD* = 0.17; see Figure 3 for a frequency distribution). The majority of participants’ language network was left-lateralized (78%) and the minority was bilateralized (22%); none was right-lateralized.

### Linear Regressions

First, the distributions of residuals for all 178 regressions were visually inspected to ensure normality, and a representative distribution can be found in Figure S1 in the Supplementary Materials. Univariate linear model fitting identified nine significant features to predict LI: one demographic (sex), three microstructural characteristics of the right hemisphere (AF density and fractional anisotropy, and frontal anterior part of the CC density), four macrostructural characteristics of the left hemisphere (FAT diameter, volume, surface area and elongation), as well as one macrostructural characteristic of the frontal anterior part of the CC (elongation). However, no univariate variable survived FDR correction for multiple comparisons (Table 1). The fact that a variable does not survive the multiple comparison correction does not mean that it has no role in predicting the LI. So, instead of assessing the predictive power of each variable individually and given the tendency of some variables to reach significance, the predictive power of the variables was tested as a group.

**Table 1.** Univariate linear model fitting

Predictors			Coefficients	*p* values	FDR *p* values
Sex			0.045	<0.001	0.131
AFD	AF	R	−0.336	0.007	0.452
Diameter	FAT	L	0.268	0.009	0.452
Volume	FAT	L	0.588	0.010	0.452
Elongation	CC_Fr1		−0.033	0.022	0.773
AFD	CC_Fr1	R	−0.932	0.031	0.923
Surface area	FAT	L	<0.001	0.041	0.923
FA	AF	R	−0.595	0.045	0.923
Elongation	FAT	L	−0.054	0.047	0.923

*Note*. Predictors are named by metrics (apparent fiber density = AFD; fractional anisotropy = FA), then tract (arcuate fasciculus = AF; frontal aslant tract = FAT; frontal anterior part of the corpus callosum = CC_Fr1), and laterality (left = L; right = R), and *p* values are corrected for false discovery rate (FDR).

To test if a set of parameters could predict LI, a regularized multiple regression linear model was performed using elastic net regression. This method also allowed us to effectively deal with both multicollinearity (see Figure S2 of Supplementary Materials for a correlation matrix) and feature selection. The Model 1 (*α* = 1, *r* = 0.13, RMSE = 0.16) based on demographic and both hemispheres data identified three most contributing predictors (Table 2): one demographic (sex) and two macrostructural characteristics (volume of the left FAT and elongation of the frontal anterior part of the CC). Model 2 utilized demographic and left hemisphere data to predict LI (*α* = 0.8, *r* = 0.10, RMSE = 0.16), highlighting five key predictors (Table 2): one demographic variable (sex) and four macrostructural characteristics of the left hemisphere—the average length of the ILF and FAT, as well as the volume of the FAT and the frontal-posterior segment of the CC. Model 3 predicted LI using demographic and right hemisphere data (*α* = 1, *r* = 0.20, RMSE = 0.16), and highlighted only one important predictor which was demographic (sex; Table 2). See Table 2 for variables’ coefficients.

**Table 2.** Regularized multiple regression model

Model 1 (*r* = 0.13; RMSE = 0.16)			Coefficients
Sex			0.003
Volume	FAT	L	0.005
Elongation	CC_Fr1		−0.005
Model 2 (*r* = 0.10; RMSE = 0.16)			Coefficients
Sex			0.009
Average length	ILF	L	−0.035
Average length	FAT	L	0.046
Volume	FAT	L	0.102
Volume	CC_Fr2	L	−0.006
Model 3 (*r* = 0.20; RMSE = 0.16)			Coefficients
Sex			0.005

*Note*. Regularized multiple regression model fitting using elastic net with demographic and (Model 1) left and right neuroimaging data, (Model 2) left hemisphere neuroimaging data, and (Model 3) right hemisphere neuroimaging data. Predictors are named by metrics, then tract (frontal aslant tract = FAT; frontal anterior part of the corpus callosum = CC_Fr1; inferior longitudinal fasciculus = ILF; frontal-posterior part of the corpus callosum = CC_Fr2), and laterality (left = L). Other abbreviations: *r* = Pearson correlation between predicted and actual values; RMSE = root-mean-square error.

### Random Forest

**Figure 4.**
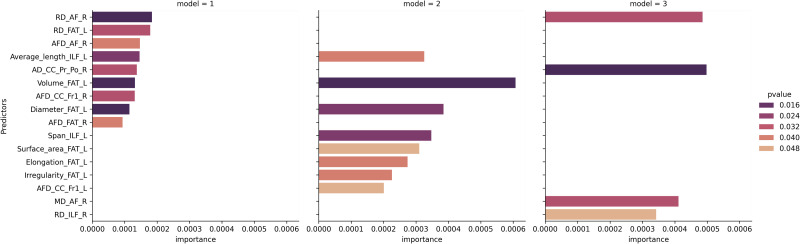
Importance of predictors in each random forest model using demographic and left and right neuroimaging data (Model 1), demographic and left hemisphere neuroimaging data (Model 2), and demographic and right hemisphere neuroimaging data (Model 3). Predictors are named by metrics (radial diffusivity = RD; apparent fiber density = AFD; axial diffusivity = AD; mean diffusivity = MD), then tract (arcuate fasciculus = AF; frontal anterior part of the corpus callosum = CC_Fr1; frontal aslant tract = FAT; inferior longitudinal fasciculus = ILF; pre/post central gyri of the corpus callosum = CC_Pr_Po) and laterality (left = L; right = R).

Model 1 (RMSE = 0.17), combining demographic and micro- and macrostructural characteristics of the left and right hemispheres, highlighted nine significant (permutation *p* values ≤ 0.05) predictors out of 178 (Table 3): six associated with microstructural measures including radial diffusivity (right AF and left FAT), axial diffusivity (right pre/post central gyri part of the CC) and density (right AF, FAT, and frontal anterior part of the CC), and three macrostructural measures such as average length (left ILF), volume (left FAT), and diameter (left FAT). The three most important predictors of LI were microstructural characteristics of the AF and FAT (Figure 4).

**Table 3.** Random forest significant predictors

Model 1 (RMSE = 0.17)			Importance (× 10^−4^)	*p* values
RD	AF	R	1.844	0.010
RD	FAT	L	1.788	0.030
AFD	AF	R	1.470	0.040
Average length	ILF	L	1.460	0.020
AD	CC_Pr_Po	R	1.374	0.030
Volume	FAT	L	1.320	0.010
AFD	CC_Fr1	R	1.315	0.030
Diameter	FAT	L	1.147	0.010
AFD	FAT	R	0.935	0.040
Model 2 (RMSE = 0.17)			Importance (× 10^−4^)	*p* values
Volume	FAT	L	6.072	0.010
Diameter	FAT	L	3.852	0.020
Span	ILF	L	3.474	0.020
Average length	ILF	L	3.256	0.040
Surface area	FAT	L	3.102	0.050
Elongation	FAT	L	2.742	0.040
Irregularity	FAT	L	2.262	0.040
AFD	CC_Fr1	L	2.010	0.050
Model 3 (RMSE = 0.17)			Importance (× 10^−4^)	*p* values
AD	CC_Pr_Po	R	4.977	0.010
RD	AF	R	4.857	0.030
MD	AF	R	4.116	0.030
RD	ILF	R	3.426	0.050

*Note*. Random forest significant (permutation *p* values ≤ 0.05) predictors using demographic and (Model 1) left and right neuroimaging data, (Model 2) left hemisphere neuroimaging data, and (Model 3) right hemisphere neuroimaging data. Predictors are named by metrics (radial diffusivity = RD; apparent fiber density = AFD; axial diffusivity = AD; mean diffusivity = MD), then tract (arcuate fasciculus = AF; frontal aslant tract = FAT; inferior longitudinal fasciculus = ILF; pre/post central gyri part of the corpus callosum = CC_Pr_Po; frontal anterior part of the corpus callosum = CC_Fr1) and laterality (left = L; right = R). RMSE = root-mean-square error.

Model 2 (RMSE = 0.17), combining demographic and neuroimaging data of the left hemisphere, revealed eight significant (permutation *p* value ≤ 0.05) predictors out of 74 (Table 3): one microstructural measure (density of the left frontal anterior part of the CC) and seven macrostructural measures including five in the left FAT (volume, diameter, surface area, elongation and irregularity) and two in the left ILF (span and average length). The two most important predictors of LI were macrostructural characteristics of the FAT (Figure 4).

Model 3 (RMSE = 0.17), including demographics and neuroimaging data of the right hemisphere, resulted in four significant (permutation *p* value ≤ 0.05) predictors out of 74 (Table 3): all were microstructural characteristics including two in the right AF (radial and mean diffusivity), one in the right ILF (radial diffusivity) and one in the right pre/post central gyri part of the CC (axial diffusivity). The most important predictor of LI was a microstructural characteristic of the CC (Figure 4).

## DISCUSSION

The objective of the present study was to determine the relationship between characteristics of white matter bundles mainly involved in the language network and rs-fMRI LI based on a seed in the left opIFG to identify potential structural indicators of LI. By using different regressions as well as machine learning algorithms, we addressed the unsolved question of whether structural characteristics of white matter bundles can be considered proxies of language laterality and gathered crucial information on this complex interaction. In the following sections, the results will be discussed separately for each bundle.

### Arcuate Fasciculus

The AF is well known for its clear role in processing auditory details, mapping sound to motor actions, and more generally, phonological processing, especially in production and this, in the left hemisphere tract (Shekari & Nozari, 2023). Our results suggest a relationship between the AF and functional language lateralization, as reported in previous studies (James et al., 2015; Ocklenburg et al., 2013; Propper et al., 2010; Sreedharan et al., 2015; Vassal et al., 2016; Vernooij et al., 2007). Our results align with other studies that have also reported a link between right AF density and LI (James et al., 2015; Vernooij et al., 2007). Interestingly, our random forest prediction models indicate that the right AF may play a greater role in predicting LI than its left counterpart, as only features of the right AF emerged as significant predictors of LI. In contrast with previous studies suggesting a correlation between AF volume and LI (Sreedharan et al., 2015; Yazbek et al., 2021), our random forest prediction models indicate that only microstructural (density, radial, and mean diffusivities), rather than macrostructural, characteristics of the AF contributed to LI prediction. This encourages future studies to further explore the relationship between the right AF microstructural characteristics and LI.

### Inferior Longitudinal Fasciculus

Our results suggest that both microstructural and macrostructural features of the ILF can help predict LI. More precisely, our regularized multiple regression and random forest models identified the radial diffusivity of the right ILF as well as the average length and the span of the left ILF as predictors of LI. However, despite having a larger sample size (*n* = 618), the present study failed to replicate the findings of James and colleagues (2015; *n* = 57) on the relationship between left and right ILF density and LI. This may be due to differences in the calculation of LI. For example, as recommended by Bradshaw and collaborators (2017a), we used signal magnitude, which is thought to provide a more robust and reliable measure, whereas James and colleagues (2015) used signal extent.

### Frontal Aslant Tract

Unexpectedly, the left FAT was among the most important predictors of LI. Indeed, the elastic net and random forest models highlighted the contribution of mainly macrostructural characteristics of the left FAT. Although after correction for multiple comparisons, volume, diameter, surface area and elongation of the left FAT did not emerge as significant predictors of LI in the univariate analyses, these four variables all returned together as main predictors in the random forest models. Left FAT volume was also a significant predictor in elastic net models, as was the average length. Random forest models highlighted other FAT macrostructural features, such as the left irregularity, but also microstructural ones, such as the radial diffusivity of the left FAT and apparent fiber density of the right FAT. Those results are not often reported most likely because only two other studies (Al Busaidi et al., 2023; Andrulyte et al., 2024) have focused on this tract and functional language lateralization. In one of these studies, connectomic analysis of quantitative anisotropy and shape feature analyses included the FAT (Andrulyte et al., 2024). This study did not identify the FAT as one of the white matter tracts allowing to predict lateralization quotient measured with tb-fMRI, neither in the connectomic analysis nor the shape analyses. In the other one, the researchers found no relationship between FAT volume asymmetry and LI (Al Busaidi et al., 2023). While we found a significant contribution of left FAT volume, our results do not support any contribution from macrostructural features of its right homonym. This suggests that the left hemisphere tract volume’s influence may have been masked when using a ratio-based approach (Al Busaidi et al., 2023). This strongly encourages future studies focussing on white matter tracts and LI to examine fasciculi from the two hemispheres separately rather than solely in a ratio. Both tracts have been associated with the domain general role in planning, timing, and coordination of sequential motor movements, with the left FAT being more specialized in speech actions, while the right FAT is proposed to be specialized in the general action control of the organism, especially in the visuospatial domain (Dick et al., 2019). Our results argue in favor of including the FAT in future studies of language lateralization to better understand its role in the language network organization.

### Corpus Callosum

The present results are in line with previous literature highlighting relationships between CC and functional language lateralization (Andrulyte et al., 2024; Häberling et al., 2011; Josse et al., 2008; Karpychev et al., 2022; Westerhausen et al., 2006). Indeed, as suggested by the study of Karpychev and colleagues (2022), one of our regularized multiple regression models indicates that CC volume is a significant predictor of LI. Thanks to our methodological choices, we were able to identify that it is perhaps more specifically the left part of the frontal-posterior segment of the CC volume that participates in the prediction of LI. In the present study, the segmentation of the CC into five subregions (frontal-anterior, frontal-posterior, pre/post central gyrus, parietal, and occipital) allowed for a greater precision regarding which parts of the bundle contribute to the prediction of LI according to different metrics. We were thus able to illustrate that certain metrics may be better suited to predict LI for certain segments of the CC only, and not for others. In addition to the metrics of the CC already identified as related to LI in previous studies (Andrulyte et al., 2024; Häberling et al., 2011; Josse et al., 2008; Karpychev et al., 2022; Westerhausen et al., 2006), our results suggest that apparent fiber density (frontal anterior part of CC in the right hemisphere), axial diffusivity (pre/post central gyrus of CC in the right hemisphere), and elongation (frontal anterior part of CC) may also be important in understanding functional language lateralization. However, we did not replicate the relationship between CC fractional anisotropy (Häberling et al., 2011) nor surface area (Josse et al., 2008), and LI. This may be explained by methodological differences, such as the fact that we divided the CC into its left and right components to isolate structural indicators based on a single hemisphere. The relationship between CC fractional anisotropy as well as surface area and LI may exist only with the whole CC (not segmented into five or divided into left and right components). Häberling and colleagues (2011) also found no significant results when segmenting the CC into three subregions (prefrontal cortices, motor cortices, parietal/temporal/occipital cortices). Other studies also failed to identify a relationship between fractional anisotropy (Karpychev et al., 2022; Perlaki et al., 2013) or surface area (Westerhausen et al., 2006) and LI. Interestingly, Westerhausen and colleagues (2006) also segmented the CC (genu, truncus, and posterior third of the CC). As the different CC segments rely on different lobes, bundle segmentation may provide a better understanding of the structure–function correspondence of the CC, but further research is needed to determine when to segment or not to segment the CC.

### Relation Between Tracts and Metrics

Overall, our results show that there is no perfect predictor of LI, either in terms of tract or metric. Depending on the bundle, different types of metrics may be appropriate as predictors (and vice versa). As each white matter tract has a different morphology, some metrics may be more descriptive for some tracts and less so for others. For example, diameter may be more appropriate for a more cylindrical-shaped tract, and curvature for a more curved one. We therefore acknowledge that the diameter of the left FAT as a significant predictor of LI must be interpreted with caution, as the FAT is a rather curved tract. The exploratory nature of our study enabled us to include numerous tracts and metrics and illustrate the variability of the combination that can be used to predict LI. Future studies, while continuing to include various metrics and tracts, could separate analyses by metric or tract to clarify the role of each independently, which goes beyond the scope of the present study. We have made our data available (https://osf.io/enjyd/files/osfstorage?view_only=3a2a19becd4c496b800df5512b09fbb2) so that these additional analyses could be carried out by future interested parties.

### Demographic Implications

In addition to neuroimaging data, demographics may influence functional language lateralization. In our study, sex was a significant predictor of LI in elastic net models. It was even the only predictor in the regularized multiple regression fitting based on demographics and right hemisphere characteristics. This could provide support for future studies to explore the relationship between structural white matter and LI in men and women separately.

### Left vs. Right Hemisphere Models

One aim of the present study was to compare predictive models based on left-hemisphere or right-hemisphere data only. By doing so, any LI predictor could then be used in the context of damage in one hemisphere to infer pre-injury language lateralization and explore its role in language recovery. Whether using elastic net or random forest modeling, all mean errors were comparable (RMSE of 0.16 or 0.17). This indicates that potential structural proxy of functional language lateralization may be found not only in the left, but also in the right hemisphere. However, considering that our LI varies from −0.11 to 0.90, an average error of 0.16 or 0.17 (16%–17% of the total range) is rather large. Also, Pearson’s correlation coefficients were all indicative of a low degree between predicted and actual values in the elastic net models. This means that predictions based on demographic information and structural white matter characteristics of the left or right hemispheres are somewhat limited. Thus, bundle features may not represent enough information to be used as proxies and other features, for example, gray matter structures from left as well as right hemispheres, should also be included in future predictive models to obtain a more accurate estimation of functional language lateralization.

### LI Using Other rs-fMRI Language Network Seeds

In the present article, the focus was made on LI using a seed in the IFG. As stated in Rolinski and collaborators (2020), Broca’s area (corresponding to the opercular and triangular parts of the IFG) was chosen as the main seed of interest based on its involvement in expressive language functioning, in addition to several studies supporting its temporal reliability (Tomasi & Volkow, 2012; Zhu et al., 2014) and lateralizing effects in resting state fMRI (Doucet et al., 2015; Tomasi & Volkow, 2012; Zhu et al., 2014). However, analyses were carried out on all seeds included in Battistella and colleagues’ work (2020). Overall, only the participant’s sex was a recurrent predictor, being even the only significant (*p*_FDR_ < 0.05) predictor in one univariate regression (see Table S4 in Supplementary Materials). Thus, depending on the seed, the most important predictors among the neuroimaging data changed. This led us to be ambivalent regarding the use of the predictors presented above in a clinical context to predict language deficits, as no tract or metric stood out as an important predictor, regardless of the seed employed.

### Methodological Considerations

The present study has some limitations and considerations. First, although the quality assessment was carried out as objectively as possible, the exclusion of a large number of FAT data (21% left FAT, 18% right FAT) may have influenced the results by removing some of the variability to those tracts that are thought to have high interindividual variability (Linn et al., 2024). Second, we are aware that white matter tracts are composed of multiple components relying on different regions of the cortex, and that multiple processes, some language-related and some not, can be associated to a tract. To account for this complexity, we segmented the CC into five segments. To maintain consistency with the studies that guided our bundle selection, we did not segment the AF, ILF, or FAT. However, we acknowledge that this may be a simplification. Third, certain methodological choices limit the generalizability of the results: Even if language tb-fMRI patterns are comparable to rs-fMRI language network (Battistella et al., 2020), the results may not be generalizable to task-based LI or to LI computed with different resting-state seeds. Also, there are inherent limitations to seed-based rs-fMRI. Although it procures clinical advantages compared to tb-fMRI, resting-state functional connectivity patterns are confined to brain regions functionally connected to the seed, and seed selection is dependent of the user choice. Also, as recommended in the mapping of language (Kumar et al., 2024), we used a minimum of 6-min. acquisition time (HCP dataset includes four runs of 15 min. each). Thus, we assumed that functional connectivity remained static across the whole duration of the rs-fMRI runs. Nonetheless, as stated by Rolinski and colleagues (2020), failure to account for dynamic changes across a range of timescales may lead to an oversimplification of the observed network findings. Fourth, although we controlled for multiple comparisons where appropriate, this study was exploratory and the results should then be replicated in another dataset with specific preliminary hypotheses. Finally, as the performances of the prediction models were low, conclusions drawn from the results must be made with caution.

### Conclusion

The main purpose of the study was to identify structural indicators of rs-fMRI LI using tractography on white matter tracts of each hemisphere separately. This objective has been partially achieved, as we have highlighted significant predictors of LI, but they are not sufficiently representative to be used as reliable proxies of functional language lateralization.

The present study contributes to a better understanding of the involvement of the dominant hemisphere and the hemisphere not traditionally associated with language (i.e., the right hemisphere) in predicting functional language lateralization. Thus, it provides a basis for future studies on the identification of a single-hemisphere-based indicator of LI that could be used in a clinical population (e.g., stroke or tumor patients).

## FUNDING INFORMATION

Marie-Ève Desjardins, Canadian Institutes of Health Research scholarship, Award ID: RN489418 - 494210. Marie-Ève Desjardins, Fonds de Recherche du Québec scholarship, Award ID: #298601 & #311549. Simona Maria Brambati, Natural Sciences and Engineering Research Council of Canada (https://dx.doi.org/10.13039/501100000038), Award ID: PVX20965-(RGP). Simona Maria Brambati, Courtois Foundation (https://dx.doi.org/10.13039/501100021783).

## AUTHOR CONTRIBUTIONS

**Marie-Ève Desjardins**: Conceptualization: Equal; Formal analysis: Lead; Funding acquisition: Equal; Methodology: Equal; Project administration: Equal; Visualization: Lead; Writing – original draft: Lead; Writing – review & editing: Lead. **Karine Marcotte**: Supervision: Supporting; Writing – review & editing: Supporting. **Xanthy Lajoie**: Methodology: Supporting; Writing – review & editing: Supporting. **Christophe Bedetti**: Data curation: Supporting; Formal analysis: Supporting; Methodology: Supporting; Software: Supporting; Visualization: Supporting; Writing – review & editing: Supporting. **Bérengère Houzé**: Funding acquisition: Supporting; Methodology: Supporting; Writing – review & editing: Supporting. **Abdelali Filali-Mouhim**: Formal analysis: Supporting; Methodology: Supporting; Validation: Supporting; Writing – review & editing: Supporting. **Arnaud Boré**: Methodology: Supporting; Software: Supporting; Writing – review & editing: Supporting. **Maxime Descoteaux**: Methodology: Supporting; Software: Supporting; Writing – review & editing: Supporting. **François Rheault**: Methodology: Supporting; Software: Supporting; Writing – review & editing: Supporting. **Simona Maria Brambati**: Conceptualization: Equal; Formal analysis: Supporting; Funding acquisition: Equal; Methodology: Supporting; Project administration: Equal; Supervision: Lead; Writing – original draft: Supporting; Writing – review & editing: Supporting.

## DATA AND CODE AVAILABILITY STATEMENT

The data that support the findings of this study are openly available in Human Connectome Project at https://www.humanconnectome.org/study/hcp-young-adult/document/extensively-processed-fmri-data-documentation (Van Essen et al., 2012). All code, databases, and order of the variables in the correlation matrix associated with the current submission are available at https://osf.io/enjyd/.

## Supplementary Material


