# Supplementary material for: Exploring the Relationship Between White Matter Tracts and Resting-State Functional Language Lateralization Index
Source: Neurobiol Lang (Camb). 2025 Jun 18;6:nol_a_00167. doi: 10.1162/nol_a_00167 (PMC12204735; doi:10.1162/nol_a_00167)
Supplement: Supplementary file 1 [file nol-6-1-167-s001.pdf]

Supplementary Material

Rs-fMRI Functional Connectivity Analyses

The second seed was defined in a portion of the planum temporal/parietal operculum and is a subportion of the temporo-parietal area (hereafter referred to as Spt; MNI coordinates: x = -51, y = -42, z = 21); the third seed was placed in the left anterior middle temporal gyrus (aMTG; MNI coordinates: x = -60, y = -6, z = -18); and the fourth seed was defined in the left posterior inferior temporal gyrus (pITG; MNI coordinates: x = -54, y = -52, z = -10).

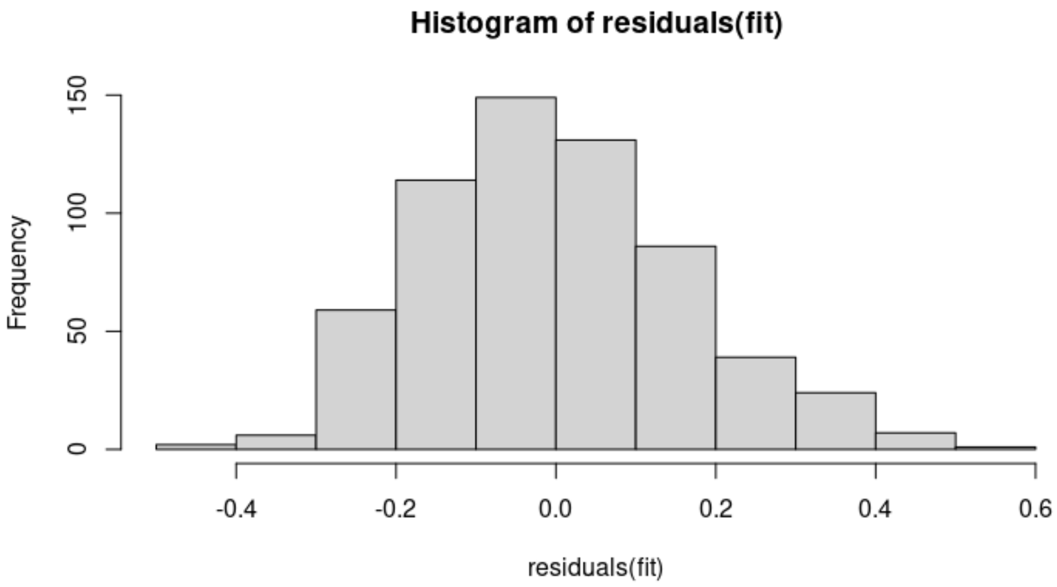

Figure 1. Representative distribution of the residuals for one univariate linear regression based on opIFG seed.

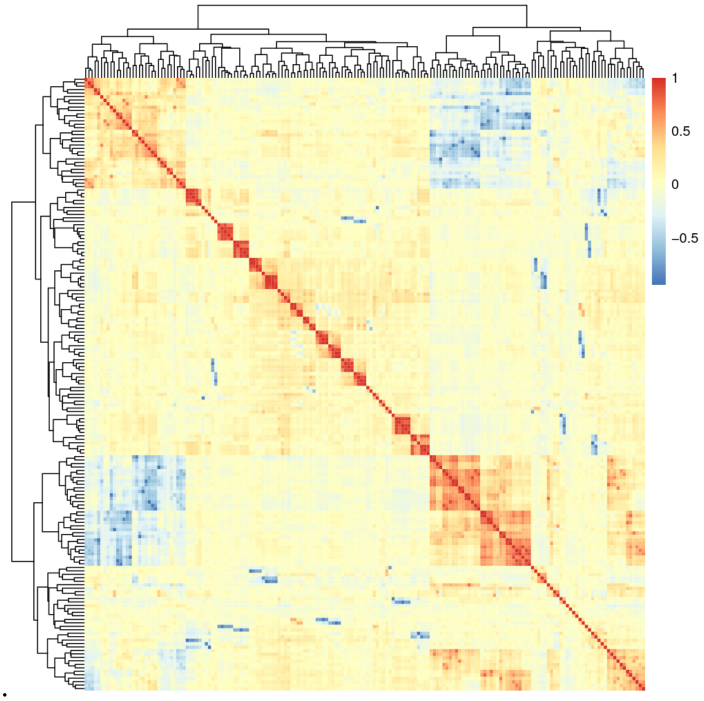

Figure 2. Correlation matrix of all demographic and neuroimaging predictors.

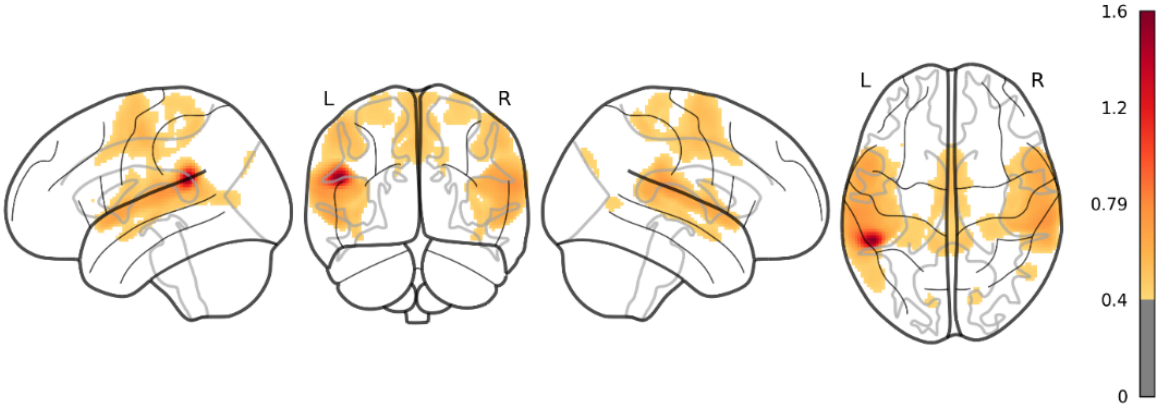

Figure 3. Mean correlation maps (z-scores transformed) of the rs-fMRI language network obtained from the Spt seed. A threshold of z=0.4 was applied to facilitate visualization.

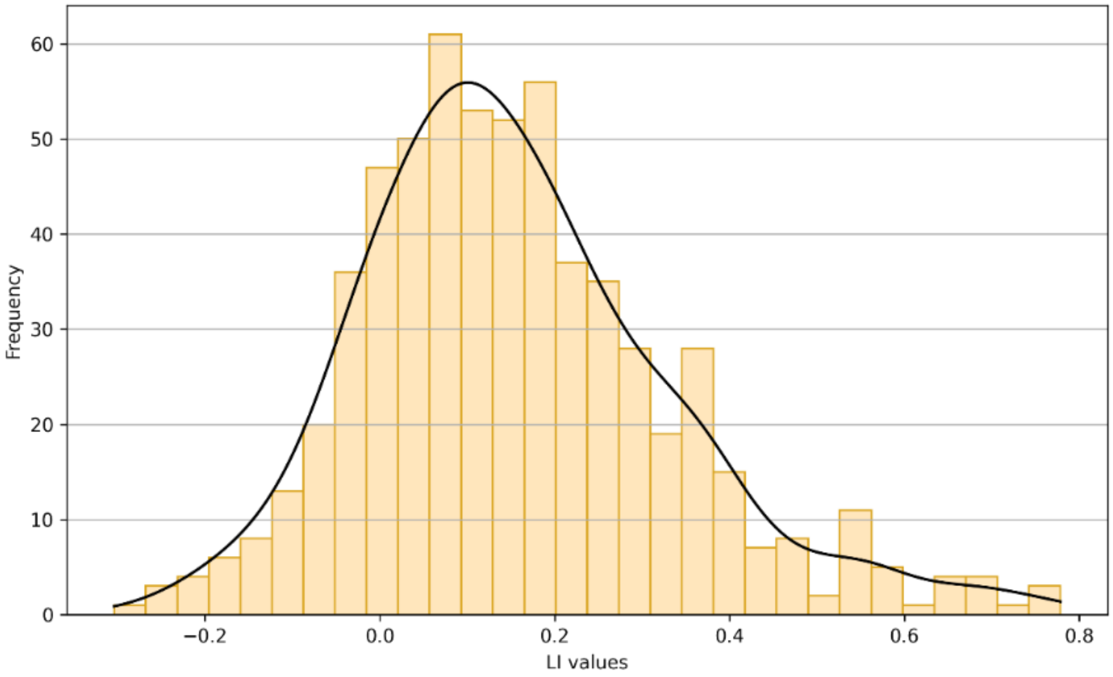

Figure 4. Frequency distribution of functional language lateralization indexes (LI) obtained with the Spt seed.

Predictors			Coefficients	*p* values	FDR *p* values
		Age	0.004	0.026	0.864
Surface area	FAT	L	0.001	0.032	0.864
Volume	CC_Pr_Po	R	0.362	0.034	0.864
		Sex	0.030	0.035	0.864
Volume	FAT	L	0.495	0.045	0.864
Diameter	FAT	L	0.219	0.048	0.864

Table 1. Univariate linear model fitting (Spt seed). Predictors are named by metrics, then tract (frontal aslant tract=FAT; pre/post central gyri part of the corpus callosum=CC_Pr_Po) and laterality (left=L; right=R), and *p* values are corrected for false discovery rate (FDR).

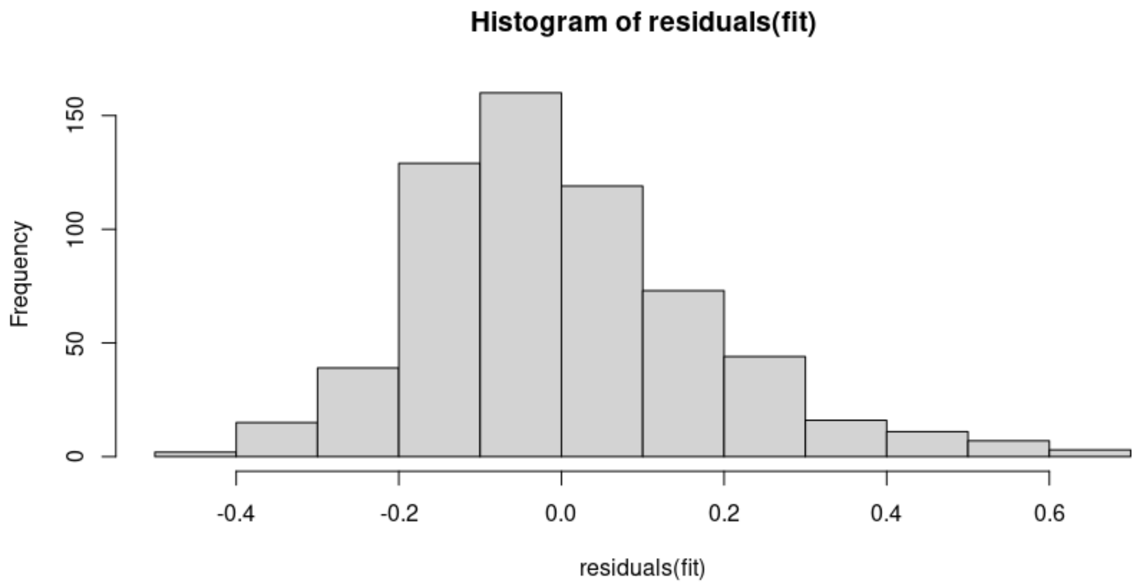

Figure 5. Representative distribution of the residuals for one univariate linear regression based on Spt seed.

a) Model 1 (*r*=-0.07; RMSE=0.19)			**Coefficients**
Diameter	FAT	L	0.082
AD	FAT	R	10.729
b) Model 2 (*r*=-0.06; RMSE=0.19)			**Coefficients**
Diameter	FAT	L	0.107
Surface area	FAT	L	<0.001
c) Model 3 (*r*=-0.07; RMSE=0.19)			**Coefficients**
Span	FAT	R	0.041
AD	FAT	R	31.793

Table 2. Regularized multiple regression model fitting (Spt seed) using elastic net with demographic and a) left and right neuroimaging data, b) left hemisphere neuroimaging data and c) right hemisphere neuroimaging data. Predictors are named by metrics (axial diffusivity=AD), then tract (frontal aslant tract=FAT) and laterality (left=L; right=R). Other abbreviations: *r*=Pearson correlation between predicted and real values; RMSE=root mean squared error.

a) Model 1 (RMSE=0.18)			**Importance** (x10^{-4})	***p* values**
FA	CC_Oc	R	2.947	0.030
AD	FAT	L	2.863	0.010
AFD	AF	R	2.816	0.010
FA	CC_Fr2	L	2.142	0.050
Surface area	CC_Fr1		2.117	0.010
Volume	CC_Pr_Po	L	1.996	0.030
Elongation	AF	L	1.722	0.040
Irregularity	AF	R	1.558	0.030
MD	FAT	R	1.508	0.050
AD	AF	L	1.367	0.050
Elongation	CC_Pr_Po		1.223	0.040
Average length	AF	L	1.160	0.050
Curvature	FAT	R	1.152	0.020
		Income	0.524	0.040
		Education	0.506	0.040
b) Model 2 (RMSE=0.18)			**Importance** (x10^{-4})	***p* values**
na	na	na	na	na
c) Model 3 (RMSE=0.18)			**Importance** (x10^{-4})	***p* values**
MD	AF	R	7.134	0.040
AFD	AF	R	5.569	0.010
Surface area	AF	R	4.797	0.030
MD	CC_Oc	R	4.060	0.050
Volume	CC_Pr_Po	R	3.631	0.010
		Age	1.785	0.010

Table 3. Random forest (Spt seed) significant (permutation p values ≤ 0.05) predictors using demographic and a) left and right neuroimaging data, b) left hemisphere neuroimaging data and c) right hemisphere neuroimaging data. Predictors are named by metrics (apparent fibre density=AFD; axial diffusivity=AD; fractional anisotropy=FA; mean diffusivity=MD), then tract (arcuate fasciculus=AF; frontal anterior part of the corpus callosum=CC_Fr1; frontal aslant tract=FAT; frontal posterior part of the corpus callosum=CC_Fr2; occipital part of the corpus callosum=CC_Oc; pre/post central gyri part of the corpus callosum=CC_Pr_Po) and laterality (left=L; right=R). Other abbreviations: RMSE=root mean squared error; na=not applicable.

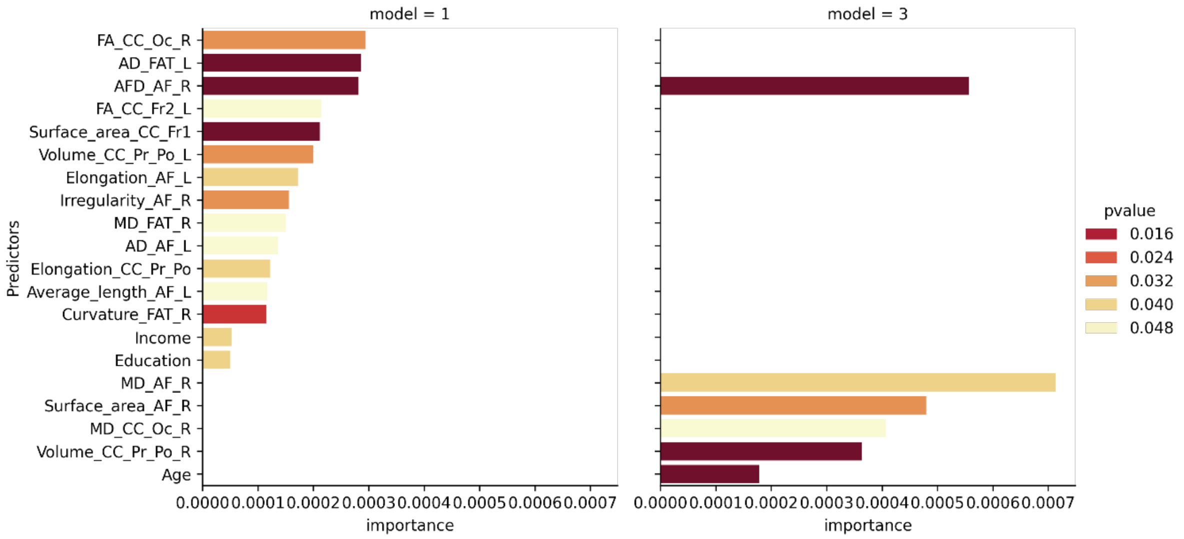

Figure 6. Importance of predictors in each random forest model (Spt seed) using demographic and left and right neuroimaging data (model 1), and demographic and right hemisphere neuroimaging data (model 3). Predictors are named by metrics (apparent fibre density=AFD; axial diffusivity=AD; fractional anisotropy=FA; mean diffusivity=MD), then tract (arcuate fasciculus=AF; frontal anterior part of the corpus callosum=CC_Fr1; frontal aslant tract=FAT; frontal posterior part of the corpus callosum=CC_Fr2; occipital part of the corpus callosum=CC_Oc; pre/post central gyri of the corpus callosum=CC_Pr_Po) and laterality (left=L; right=R). Note: model 2 (demographic and left hemisphere neuroimaging data) is not included in the figure since no predictor was significant (permutation p values ≤ 0.05).

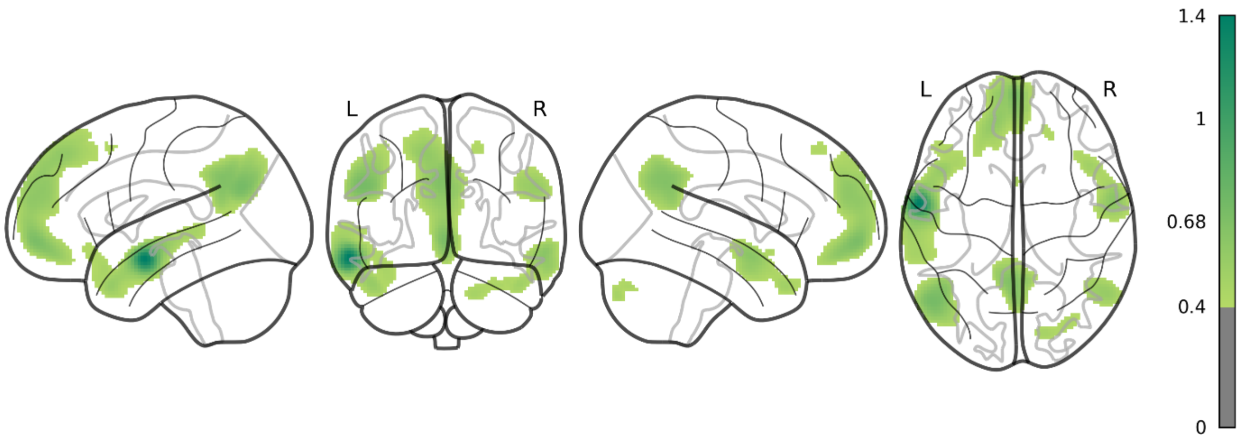

Figure 7. Mean correlation maps (z-scores transformed) of the rs-fMRI language network obtained from the aMTG seed. A threshold of z=0.4 was applied to facilitate visualization.

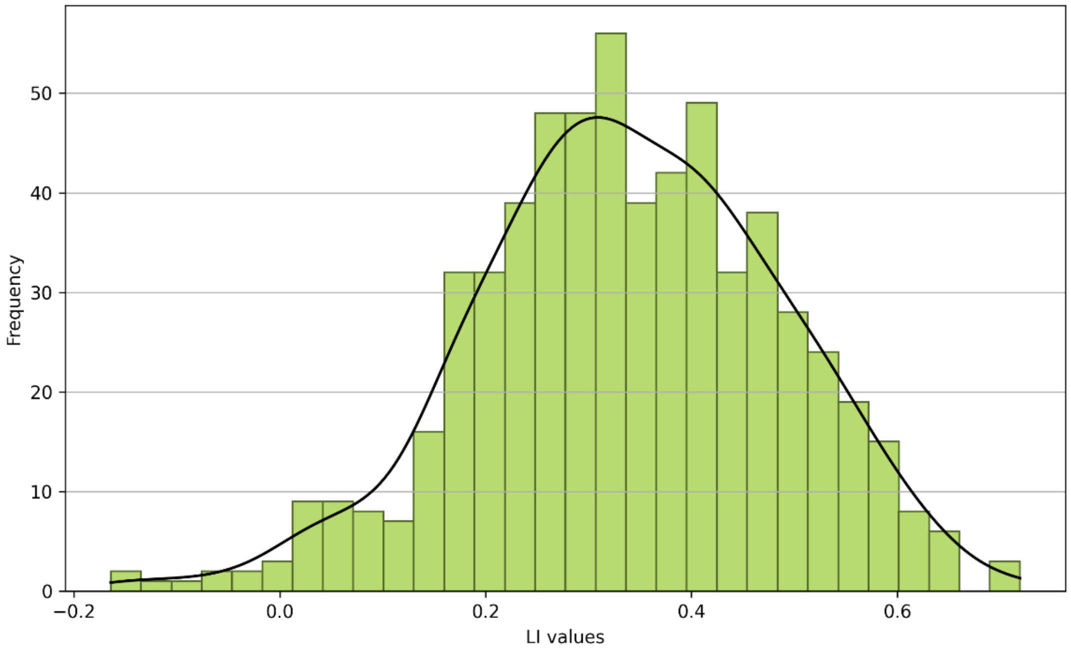

Figure 8. Frequency distribution of functional language lateralization indexes (LI) obtained with the aMTG seed.

Predictors			Coefficients	*p* values	FDR *p* values
	Sex		-0.045	<0.001	0.026
	Handedness		<0.001	0.002	0.162
Average length	CC_Fr2		-0.217	0.003	0.162
Average length	FAT	L	-0.205	0.005	0.209
AFD	CC_Fr2	L	-1,171	0.007	0.225
Span	FAT	L	-0.259	0.008	0.225
AFD	CC_Fr2	R	-1.121	0.011	0.267
FA	CC_Fr2	L	-0.602	0.014	0.292
RD	CC_Fr2	L	466.940	0.015	0.292
Volume	FAT	L	-0.483	0.018	0.315
AFD	CC_Pr_Po	L	-1.022	0.019	0.315
Surface area	CC_Fr1		<0.001	0.025	0.372
Surface area	FAT	L	>-0.001	0.039	0.531
Diameter	ILF	R	-0.165	0.046	0.531
Irregularity	CC_Fr1		0.033	0.046	0.531
Diameter	FAT	L	-0.181	0.048	0.531

Table 4. Univariate linear model fitting (aMTG seed). Predictors are named by metrics (apparent fibre density=AFD; fractional anisotropy=FA, radial diffusivity=RD), then tract

(frontal anterior part of the corpus callosum=CC_Fr1; frontal aslant tract=FAT; frontal posterior part of the corpus callosum=CC_Fr2; inferior longitudinal fasciculus=ILF; pre/post central gyri part of the corpus callosum=CC_Pr_Po) and laterality (left=L; right=R), and *p* values are corrected for false discovery rate FDR).

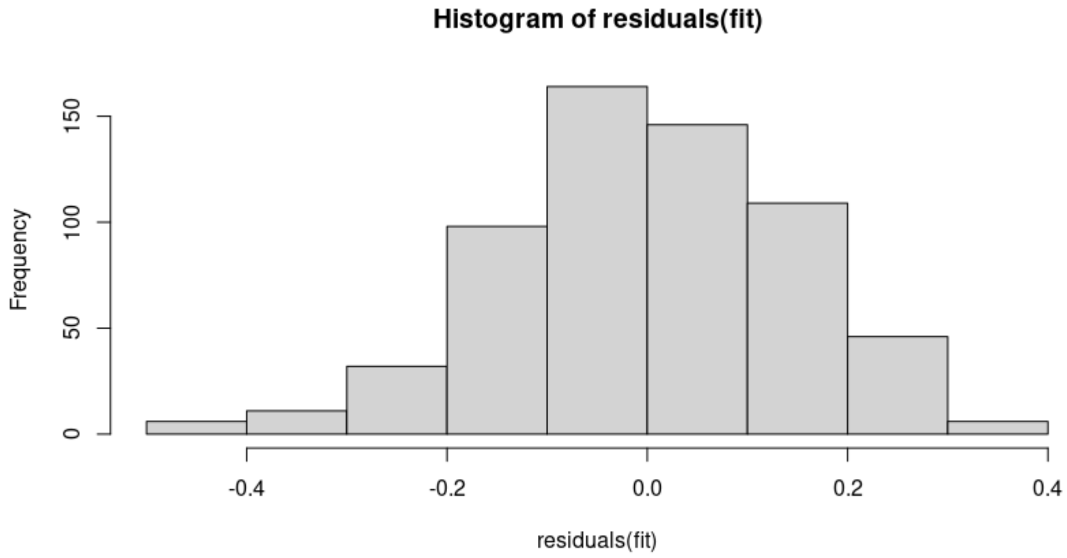

Figure 9. Representative distribution of the residuals for one univariate linear regression based on aMTG seed.

a) Model 1 (*r*=0.10; RMSE=0.15)			**Coefficients**
Averale length	CC_Fr2		-0.071
AFD	CC_Fr2	R	-0.077
		Sex	-0.019
b) Model 2 (*r*=0.21; RMSE=0.15)			**Coefficients**
		Handedness	<0.001
		Education	0.004
		Sex	-0.039
Average length	AF	L	0.023
Span	AF	L	0.005
Surface area	AF	L	<0.001
FA	AF	L	0.103
Average length	ILF	L	0.007

Span	ILF	L	0.017
Diameter	ILF	L	-0.001
Irregularity	ILF	L	0.006
Average length	FAT	L	-0.056
Span	FAT	L	-0.181
Irregularity	FAT	L	-0.001
Volume	FAT	L	-0.142
AFD	FAT	L	0.219
Volume	CC_Fr1	L	0.282
Volume	CC_Oc	L	0.342
Volume	CC_Pr_Po	L	-0.026
AD	CC_Pa	L	-98.076
FA	CC_Fr2	L	-0.356
MD	CC_Fr1	L	142.148
AFD	CC_Fr1	L	0.829
AFD	CC_Fr2	L	-1.043
AFD	CC_Oc	L	-0.034
AFD	CC_Pr_Po	L	-0.007
c) Model 3 (*r*=0.12; RMSE=0.15)			**Coefficients**
		Handedness	<0.001
		Sex	-0.023
AFD	CC_Fr2	R	-0.248

Table 5. Regularized multiple regression model fitting (aMTG seed) using elastic net with demographic and a) left and right neuroimaging data, b) left hemisphere neuroimaging data and c) right hemisphere neuroimaging data. Predictors are named by metrics (apparent fiber density=AFD; axial diffusivity=AD; fractional anisotropy=FA; mean diffusivity=MD), then tract (arcuate fasciculus=AF; frontal anterior part of the corpus callosum=CC_Fr1; frontal aslant tract=FAT; frontal posterior part of the corpus callosum=CC_Fr2; inferior longitudinal fasciculus=ILF; occipital part of the corpus callosum=CC_Oc; parietal part of the corpus callosum=CC_Pa; pre/post central gyri part of the corpus callosum=CC_Pr_Po) and laterality (left=L; right=R). Other abbreviations: *r*=Pearson correlation between predicted and real values; RMSE=root mean squared error.

a) Model 1 (RMSE=0.15)			**Importance** (x10^{-4})	***p* values**
	Handedness		1.729	0.010
RD	CC_Fr2	L	1.597	0.020
Span	FAT	L	1.484	0.010
Span	CC_Fr1		1.324	0.020
Surface area	CC_Fr1		1.310	0.050
Volume	CC_Pr_Po	R	1.060	0.040
AFD	CC_Fr2	L	0.991	0.030
Average length	CC_Pr_Po		0.893	0.050
	Sex		0.350	0.010
b) Model 2 (RMSE=0.15)			**Importance** (x10^{-4})	***p* values**
MD	CC_Fr1	L	2.604	0.030
	Handedness		2.434	0.010
Span	FAT	L	2.315	0.030
AFD	CC_Fr1	L	2.061	0.020
Curvature	ILF	L	1.687	0.020
	Sex		1.686	0.010
	Age		1.155	0.030
c) Model 3 (RMSE=0.15)			**Importance** (x10^{-4})	***p* values**
	Handedness		2.986	0.010
AFD	CC_Fr2	R	2.624	0.030
Volume	ILF	R	2.533	0.040
	Sex		2.183	0.010
Volume	AF	R	2.104	0.050
Volume	CC_Pr_Po	R	1.804	0.050

Table 6. Random forest (aMTG seed) significant (permutation p values ≤ 0.05) predictors using demographic and a) left and right neuroimaging data, b) left hemisphere neuroimaging data and c) right hemisphere neuroimaging data. Predictors are named by metrics (apparent fibre density=AFD; mean diffusivity=MD; radial diffusivity=RD), then tract (arcuate fasciculus=AF; frontal anterior part of the corpus callosum=CC_Fr1; frontal aslant tract=FAT; frontal posterior part of the corpus callosum=CC_Fr2; inferior longitudinal fasciculus=ILF; pre/post central gyri part of the corpus callosum=CC_Pr_Po) and laterality (left=L; right=R). Other abbreviations: RMSE=root mean squared error.

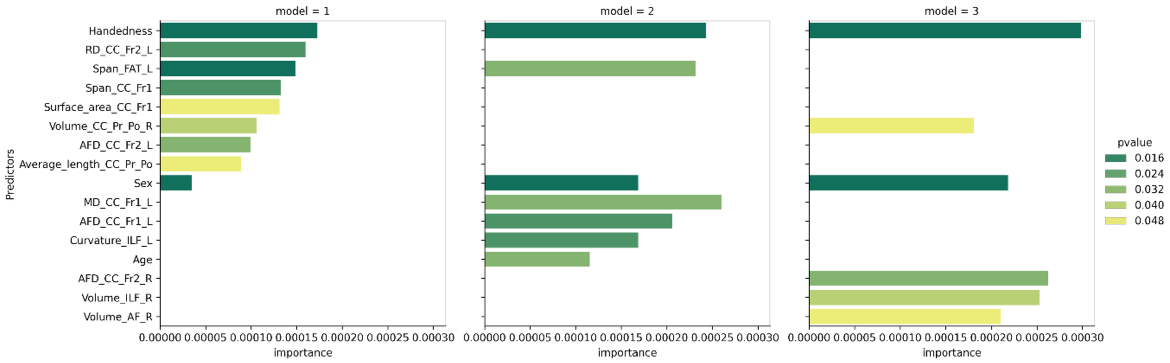

Figure 10. Importance of predictors in each random forest model (aMTG seed) using demographic and left and right neuroimaging data (model 1), demographic and left hemisphere neuroimaging data (model 2), and demographic and right hemisphere neuroimaging data (model 3). Predictors are named by metrics (apparent fibre density=AFD; mean diffusivity=MD; radial diffusivity=RD), then tract (arcuate fasciculus=AF; frontal anterior part of the corpus callosum=CC_Fr1; frontal aslant tract=FAT; frontal posterior part of the corpus callosum=CC_Fr2; inferior longitudinal fasciculus=ILF; pre/post central gyri of the corpus callosum=CC_Pr_Po) and laterality (left=L; right=R).

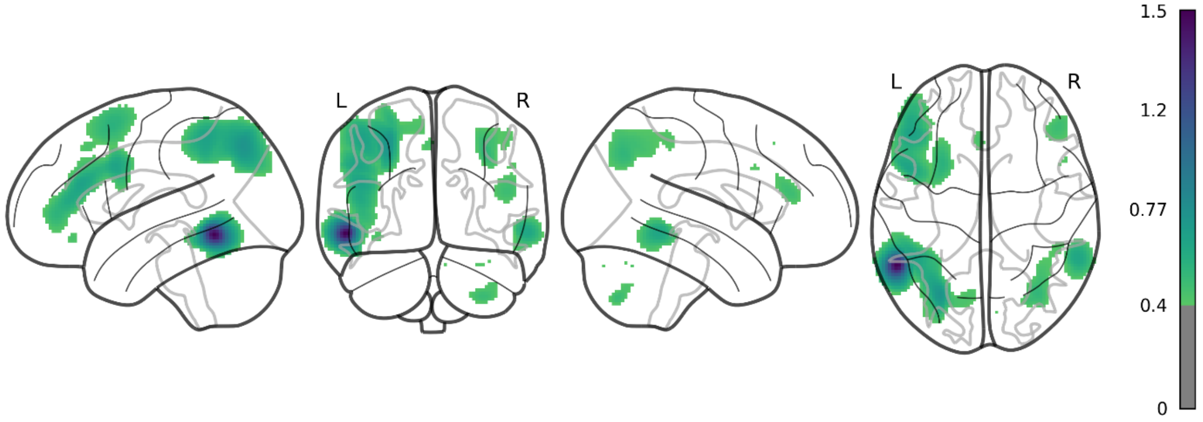

Figure 11. Mean correlation maps (z-scores transformed) of the rs-fMRI language network obtained from the pITG seed. A threshold of z=0.4 was applied to facilitate visualization.

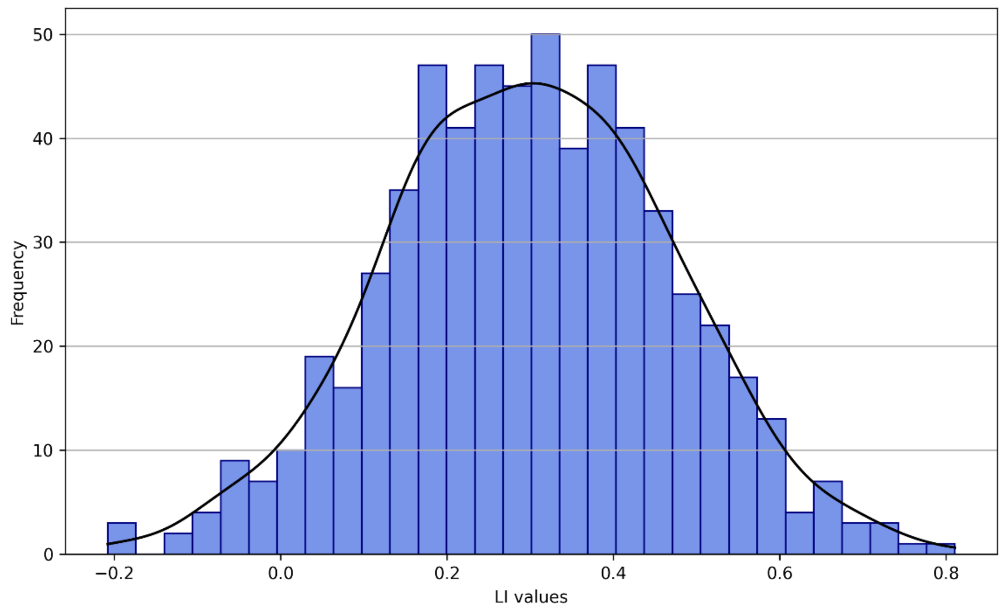

Figure 12. Frequency distribution of functional language lateralization indexes (LI) obtained with the pITG seed.

Predictors			Coefficients	*p* values	FDR *p* values
AFD	CC_Pa	L	-2.012	<0.001	0.082
AFD	CC_Fr2	R	-1.617	0.001	0.087
AFD	CC_Pr_Po	L	-1.608	0.001	0.087
AFD	CC_Pa	R	-1.708	0.003	0.112
AD	AF	L	518.795	0.003	0.112
AFD	CC_Oc	L	-0.934	0.006	0.141
		Sex	-0.037	0.007	0.141
AFD	CC_Fr2	L	-1.360	0.007	0.141
AD	FAT	R	606.097	0.008	0.141
AD	FAT	L	606.583	0.008	0.141
AFD	CC_Pr_Po	R	-1.269	0.010	0.160
MD	CC_Pr_Po	L	625.591	0.015	0.216
AD	AF	R	510.474	0.023	0.314
AD	CC_Fr1	R	371.160	0.029	0.347
Curvature	CC_Pa		0.033	0.029	0.347
AFD	ILF	L	-0.194	0.041	0.426
MD	CC_Fr2	R	484.396	0.050	0.426

Table 7. Univariate linear model fitting (pITG seed). Predictors are named by metrics (apparent fiber density=AFD; axial diffusivity=AD; mean diffusivity=MD), then tract

(arcuate fasciculus=AF; frontal anterior part of the corpus callosum=CC_Fr1; frontal aslant tract=FAT; frontal posterior part of the corpus callosum=CC_Fr2; inferior longitudinal fasciculus=ILF; occipital part of the corpus callosum=CC_Oc; parietal part of the corpus callosum=CC_Pa; pre/post central gyri part of the corpus callosum=CC_Pr_Po) and laterality (left=L; right=R), and *p* values are corrected for false discovery rate (FDR).

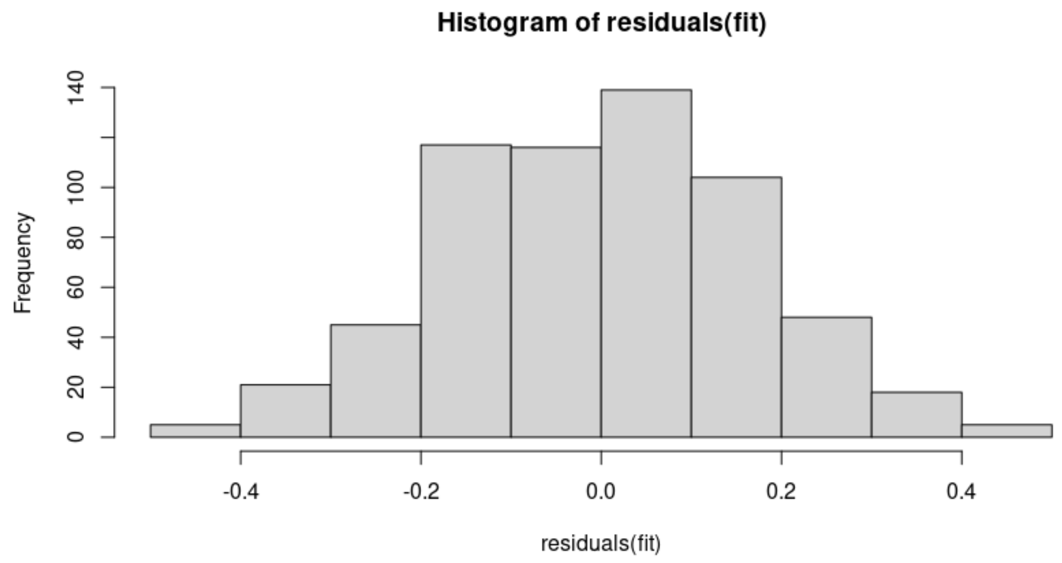

Figure 13. Representative distribution of the residuals for one univariate linear regression based on pITG seed.

a) Model 1 (*r*=0.05; RMSE=0.18)			**Coefficients**
	Handedness		<0.001
	Education		0.001
Elongation	AF	L	-0.004
Span	AF	R	0.019
Curvature	AF	R	-0.002
AFD	AF	L	0.024
AD	AF	L	81.115
Average length	ILF	L	0.007
AD	FAT	L	52.653
Elongation	CC_Fr1		-0.019
Surface area	CC_Oc		<0.001
Curvature	CC_Pa		0.002
Diameter	CC_Pa		-0.024

Volume	CC_Pa	R	-0.246
FA	CC_Oc	R	-0.054
AFD	CC_Fr2	L	-0.364
AFD	CC_Pa	L	-0.676
AFD	CC_Fr2	R	-0.011
		Sex	-0.014
b) <u>Model 2</u> (*r*=0.02; RMSE=0.18)			**Coefficients**
		Handedness	<0.001
		Education	0.004
		Sex	-0.021
Curvature	AF	L	-0.029
Elongation	AF	L	-0.006
AFD	AF	L	0.119
FA	AF	L	0.013
AD	AF	L	125.150
Average length	ILF	L	0.019
Span	ILF	L	0.025
Diameter	ILF	L	-0.041
Elongation	ILF	L	0.002
Irregularity	ILF	L	0.026
AFD	ILF	L	-0.010
Curvature	FAT	L	0.177
Irregularity	FAT	L	0.003
FA	FAT	L	0.274
AD	FAT	L	242.655
Volume	CC_Fr1	L	0.558
Volume	CC_Oc	L	0.310
Volume	CC_Pa	L	-0.537
AD	CC_Fr1	L	-171.425
AD	CC_Oc	L	-156.047
FA	CC_Fr2	L	-0.270
FA	CC_Oc	L	-0.042
MD	CC_Pr_Po	L	116.690
RD	CC_Fr2	L	25.227
AFD	CC_Fr2	L	-0.541
AFD	CC_Pa	L	-0.909
c) <u>Model 3</u> (*r*=0.03; RMSE=0.18)			**Coefficients**
		Handedness	<0.001
		Education	0.003
		Sex	-0.022
Span	AF	R	0.022
Curvature	AF	R	-0.015
Irregularity	AF	R	<0.001
AD	AF	R	98.124
AFD	ILF	R	-0.006
Volume	CC_Fr1	R	0.240

Volume	CC_Oc	R	0.041
Volume	CC_Pa	R	-0.367
FA	CC_Oc	R	-0.069
MD	CC_Fr1	R	125.748
AFD	CC_Fr1	R	-0.229
AFD	CC_Fr2	R	-0.508
AFD	CC_Pa	R	

Table 8. Regularized multiple regression model fitting (pITG seed) using elastic net with demographic and a) left and right neuroimaging data, b) left hemisphere neuroimaging data and c) right hemisphere neuroimaging data. Predictors are named by metrics (apparent fiber density=AFD; axial diffusivity=AD; fractional anisotropy=FA; mean diffusivity=MD; radial diffusivity=RD), then tract (arcuate fasciculus=AF; frontal anterior part of the corpus callosum=CC_Fr1; frontal aslant tract=FAT; frontal posterior part of the corpus callosum=CC_Fr2; inferior longitudinal fasciculus=ILF; occipital part of the corpus callosum=CC_Oc; parietal part of the corpus callosum=CC_Pa; pre/post central gyri part of the corpus callosum=CC_Pr_Po) and laterality (left=L; right=R). Other abbreviations: *r*=Pearson correlation between predicted and real values; RMSE=root mean squared error.

a) Model 1 (RMSE=0.17)			**Importance** ($x10^{-4}$)	***p* values**
AFD	CC_Fr2	R	2.797	0.010
Elongation	CC_Pa		2.426	0.010
AFD	CC_Pa	R	2.010	0.010
AD	AF	L	1.889	0.010
MD	CC_Pr_Po	L	1.729	0.030
Volume	CC_Pa	R	1.653	0.040
AFD	CC_Pa	L	1.464	0.040
RD	ILF	L	1.301	0.030
Average length	FAT	R	1.237	0.050
Elongation	ILF	L	1.126	0.030
Span	CC_Pa		1.067	0.030
AFD	AF	L	1.057	0.050
b) Model 2 (RMSE=0.17)			**Importance** ($x10^{-4}$)	***p* values**
AFD	CC_Pa	L	4.630	0.010
MD	CC_Pa	L	4.296	0.030
AFD	CC_Fr2	L	4.183	0.010
AFD	CC_Pr_Po	L	2.995	0.040

c) Model 3 (RMSE=0.17)			**Importance** (x10^{-4})	***p* values**
RD	CC_Pa	R	4.844	0.050
AFD	CC_Pa	R	4.505	0.020
AD	FAT	R	4.147	0.030
AFD	CC_Fr2	R	4.022	0.010
AFD	FAT	R	2.867	0.030
		Sex	0.472	0.030

Table 9. Random forest (pITG seed) significant (permutation *p* values ≤ 0.05) predictors using demographic and a) left and right neuroimaging data, b) left hemisphere neuroimaging data and c) right hemisphere neuroimaging data. Predictors are named by metrics (apparent fibre density=AFD; axial diffusivity=AD; mean diffusivity=MD; radial diffusivity=RD), then tract (arcuate fasciculus=AF; frontal aslant tract=FAT; frontal posterior part of the corpus callosum=CC_Fr2; inferior longitudinal fasciculus=ILF; parietal part of the corpus callosum=CC_Pa; pre/post central gyri part of the corpus callosum=CC_Pr_Po) and laterality (left=L; right=R). Other abbreviations: RMSE=root mean squared error.

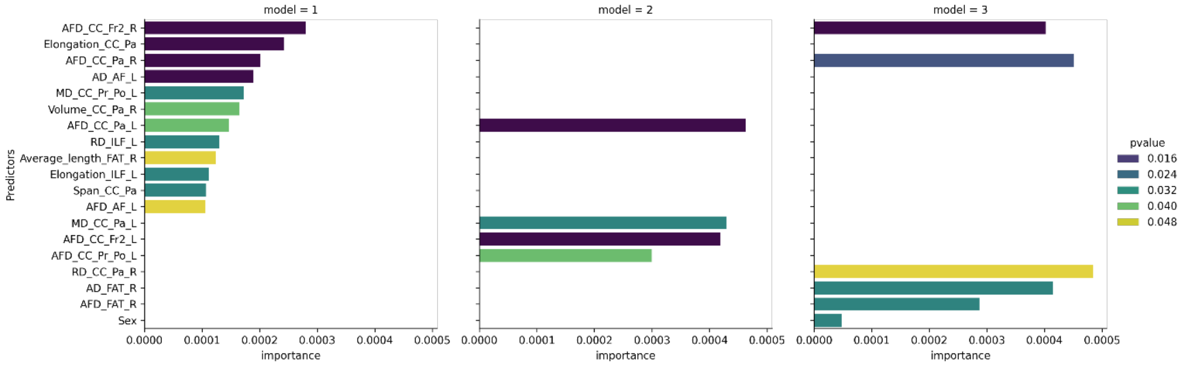

Figure 14. Importance of predictors in each random forest model (pITG seed) using demographic and left and right neuroimaging data (model 1), demographic and left hemisphere neuroimaging data (model 2), and demographic and right hemisphere neuroimaging data (model 3). Predictors are named by metrics (apparent fibre density=AFD; axial diffusivity=AD; mean diffusivity=MD; radial diffusivity=RD), then tract (arcuate

fasciculus=AF; frontal aslant tract=FAT; frontal posterior part of the corpus callosum=CC_Fr2; inferior longitudinal fasciculus=ILF; parietal part of the corpus callosum=CC_Pa; pre/post central gyri of the corpus callosum=CC_Pr_Po) and laterality (left=L; right=R).
